# dbSAP: single amino-acid polymorphism database for protein variation detection

**DOI:** 10.1093/nar/gkw1096

**Published:** 2016-11-28

**Authors:** Ruifang Cao, Yan Shi, Shuangguan Chen, Yimin Ma, Jiajun Chen, Juan Yang, Geng Chen, Tieliu Shi

**Affiliations:** The Center for Bioinformatics and Computational Biology, Shanghai Key Laboratory of Regulatory Biology, the Institute of Biomedical Sciences and School of Life Sciences, East China Normal University, Shanghai 200241, China

## Abstract

Millions of human single nucleotide polymorphisms (SNPs) or mutations have been identified so far, and these variants could be strongly correlated with phenotypic variations of traits/diseases. Among these variants, non-synonymous ones can result in amino-acid changes that are called single amino-acid polymorphisms (SAPs). Although some studies have tried to investigate the SAPs, only a small fraction of SAPs have been identified due to inadequately inferred protein variation database and the low coverage of mass spectrometry (MS) experiments. Here, we present the dbSAP database for conveniently accessing the comprehensive information and relationships of spectra, peptides and proteins of SAPs, as well as related genes, pathways, diseases and drug targets. In order to fully explore human SAPs, we built a customized protein database that contained comprehensive variant proteins by integrating and annotating the human SNPs and mutations from eight distinct databases (UniProt, Protein Mutation Database, HPMD, MSIPI, MS-CanProVar, dbSNP, Ensembl and COSMIC). After a series of quality controls, a total of 16 854 SAP peptides involving in 439 537 spectra were identified with large scale MS datasets from various human tissues and cell lines. dbSAP is freely available at http://www.megabionet.org/dbSAP/index.html.

## INTRODUCTION

Genetic variations can be divided into diverse categories including single nucleotide polymorphisms (SNPs), insertions, deletions, inversions, translocations and duplications (1), and the lengths of these variants may range from single nucleotide to gross alterations in the whole karyotype (2). The improvement of high-throughput sequencing technologies has enabled researchers to identify and characterize the genetic variants of interested species in genome-wide scale with single nucleotide resolution (3–5). The non-synonymous variants harbored in protein-coding regions can cause amino acid changes on related protein sequences, which may result in functional changes of corresponding proteins. For example, β-A myloid precursor protein mutation [alanine-673→valine] causes Alzheimer's disease in the homozygous state (6).

Technologies for protein identification are in fast revolution. Shotgun proteomics uses bottom-up proteomics techniques to identify proteins (7) in complex mixtures with a combination of high performance liquid chromatography, representing a preferred way to investigate the protein dynamics from multiple functional dimensions. For peptides or proteins detection, one of the most commonly used approaches is to search the mass spectra against a sequence database of known proteins derived from the human reference genome or cDNA sequence repositories (8), such as database searching method. Although this strategy is easy to obtain high-quality spectra at a population level, the spectra that contain variations could be missed due to the fact that corresponding amino acid changes are absent from the reference protein database.

An efficient way to identify the spectra that harbor single amino-acid polymorphisms (SAPs) is to improve the reference protein database by adding amino acid changes resulted from corresponding non-synonymous genomic variants (9), or customizing the peptide database with SNPs from dbSNP (10) and mutations from COSMIC (11). In addition, Sheynkman *et al.* have tried to use the Jurkat human cell lines to describe various characteristics of the detected SAP peptides, including their transcriptional abundance (12). Although these studies have facilitated the exploration of SAPs to certain extent, they do not focus on the variant spectra. With the rapid development of spectral library searching methods in peptide identification, a growing number of studies have paid attention to the peptides/proteins as well as the corresponding spectra, for example, the National Institute of Science of Technology (NIST), which provides a widely used resource for spectral library searching, contains over 719 338 mass spectra (3 May 2016) (13), the European Bioinformatics Institute (EBI)- PRoteomics IDEntifications (PRIDE) database also provides updated spectral library recently (14). However, the spectral library from Peptide Atlas has not been updated yet since 2013 (15). Since the detection of SAPs caused by genomic variants is crucial for understanding the functional consequence/significance of these non-synonymous variants, it is worth to further explore those SAPs with abundant public mass spectrometry (MS) data.

Here we present a comprehensive SAP database named dbSAP that catalogs the spectra harboring variants and related abundant information we collected. Firstly, we constructed a customized protein sequence database that contains a large number of corresponding amino acid changes derived from known SNPs and mutations. The comprehensive set of variations was collected from eight SNP and mutation public databases with redundancy removed according to their sequence comparison. Secondly, we built a refined workflow for variant peptide identification based on Mascot database searching software (V2.3.0) (16). Finally, we systematically identified SAPs using the MS data from 59 distinct cancer cell lines (17), Human Proteome (18,19) and NIST (20), and built a database to store SAPs, called dbSAP, we also constructed a user-friendly website to display the SAP spectral library with rich information from spectra, peptides, proteins, genes, etc.

## MATERIALS AND METHODS

### Human protein variant database construction

To build the protein database that contains human variants, we integrated the variant data from UniProt (16 April 2014) (21), PMD (26 May 2007) (22), HPMD (2012, the latest version) (23), MS-CanProVar (corresponding to Ensembl V54, the latest version) (24), MSIPI (v3.67) (25), COSMIC (v68) (11) and non-synonymous SNPs (nsSNPs) from dbSNP (dbsnp_138.hg19) (10), Ensembl (1000 Genome and HapMap, v74) (26). A total of 3 052 321 and 1 100 191 unique nsSNPs and mutations were collected to construct the theoretical protein variant database, named as theoretical SAP database which can be downloaded from the ‘Download’ webpage. The nsSNPs and mutations we collected were annotated and converted to corresponding amino acid changes using ANNOVAR (12 July 2014) (27) with gene-based annotation mode based on human reference genome hg19/GRCh37. An in-house script was used to change the reference amino acid (Aa) to the alternative Aa derived from variants after ANNOVAR annotation. Next, the protein sequences contained Aa changes were incorporated with Ensembl proteins and the redundant sequences were excluded, the resulted theoretical protein set was then used for subsequent SAP identification.

### Peptide identification

**Figure 1.**
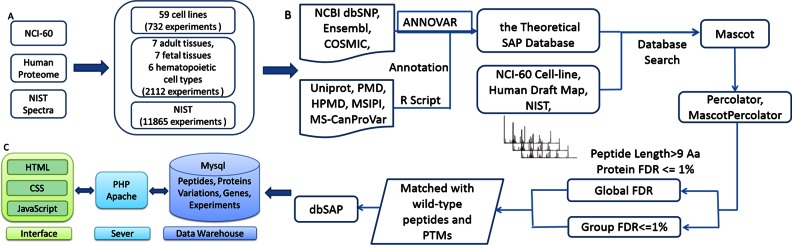
Flow chart of identifying and displaying variant peptides/proteins. (**A**) Experimental datasets used for the detection of variant peptides. (**B**) Workflow for identifying variant peptides from shotgun proteomics data. Raw uninterpreted MS/MS files were searched with Mascot against SAP database. A series of QC steps were applied to get the final variant peptides. (**C**) Storage and display structures of dbSAP.

The MS data we used for SAPs detection were from three different resources (Figure 1A): the NCI-60 panel (17), the Human Proteome (18,19) and NIST (20). The NCI-60 panel contained 59 individual cancer cell lines derived from nine distinct tissues (including breast, colon, ovarian, CNS, leukemia, lung, melanoma, prostate and renal). Raw liquid chromatography tandem-mass-spectrometry (LC MS/MS) data of human proteome were from Min-Sik Kim laboratory, including 17 different adult tissues, 7 distinct fetal tissues and 6 haematopietic cell types. A total of 11 865 MS experiments from diverse tissues of NIST repository were collected for subsequent analyses.

**Figure 2.**
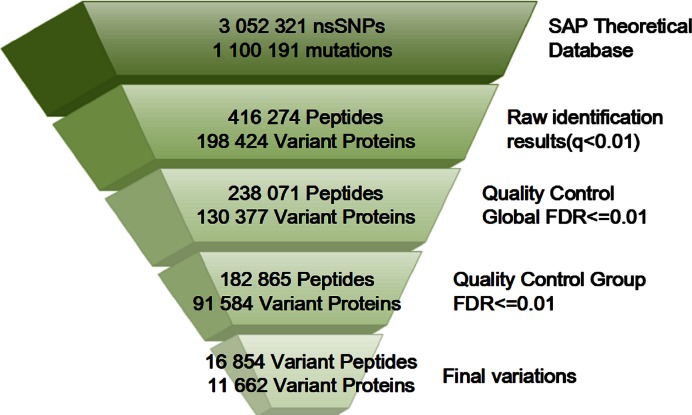
The number of peptides and proteins for SAP identification in each step of QC workflow.

All MS data were then searched against the aforementioned non-redundant variant protein database (theoretical SAP database) using Mascot database search algorithm (16), which performed a sensitive and accurate peptide and protein detection from MS data. To evaluate the false positive rate (FDR), a decoy database was generated from the theoretical SAP database using a perl script provided by Mascot. We specified Trypsin as the proteolytic enzyme and allowed up to two missing cleavages. Charge states of +2, +3 and +4 were enabled for parent ions. The error window was set to ±20 ppm on experimental peptide mass values and ±0.5 Da for MS/MS fragment ion. The overall workflow of database search was shown in Figure 1B. After Mascot database search, we totally identified 416 274 peptides and 198 424 proteins (Figure 2).

### Quality control

MascotPercolator (28) that interfaces Mascot with Percolator (29), a well performing semi-supervised machine learning method for database result rescoring, was used as the first quality control (QC) tool in our QC pipeline, the method was demonstrated to be amenable for both low and high accuracy MS data, After MascotPercolator rescoring, we employed the *q*-value of peptides to control the global FDR (criteria: ≤0.01) at protein level and obtained 238 071 peptides and 130 377 proteins (Figure 2). In addition, only the peptides that were longer than 9 Aa were screened out for SAP detection (14). Next, we mapped those identified peptides to protein sequences, only the matched peptides that contained the same variant Aa as that of in protein sequences were considered as SAP peptides preliminarily. The obtained SAP peptides were further trimmed by discarding the variant peptides which were identical to those peptides existing in the wild-type protein database. Finally, considering the high risk of false positive and low sensitivity of peptide detection in SAP-containing database searching, we took another stringent FDR control method, which can effectively adjust the FDR values obtained from previous step of corresponding method (30,31) (denoted as group FDR). The group FDR was described as bellow:

$FD{R_k}(x)$ means that the subgroup FDR of mutational peptide identifications with scores greater than x.

\begin{equation*}FD{R_k}(x) = P(F|{I_k},X >x)\end{equation*}

Where F signifies an event of false peptide identification and ${I_k}$represents an identification of a peptide carrying mutation. Then, an intermediate estimate of $FD{R_k}(x)$was derived based on Bayes rule:

\begin{equation*}\overline {FD{R_k}(x)} = \frac{{N(x)}}{{{N_k}(x)}}{\gamma _k}(x)FDR(x)\end{equation*}

Where N(x) is the number of target identifications with scores greater than x and ${N_k}(x)$is the number of target identifications of mutation with scores greater than x. ${\gamma _k}(x)$ is the probability that a spectrum would be identified as a peptide with mutation given that the identification is false and the score is greater than x. According to the observations from real data,${\gamma _k}(x)$can be approximated by a linear function of x,

\begin{equation*}\overline {{\gamma _k}(x)} = ax + b\end{equation*}

where *a* and *b* are coefficients to be determined. As a result, the final estimate of $FD{R_k}(x)$becomes$\frac{{N(x)}}{{{N_k}(x)}}(\overline a x + \overline b )FDR(x)$. This estimate is called transferred FDR for mutational peptides, indicating that it is derived from the global FDR rather than estimated completely from data. After group FDR, 182 865 peptides and 91 584 variant proteins were reserved (Figure 2).

Several factors could lead to high false positive ratio in variant peptide identification (9): (i) post-translational modifications (PTM) resulted in amino acid conversion; (ii) mistakes in discrimination of reference Aa and alternative Aa due to their close masses and vice versa (such as K→Q and L→I); (iii) cell line cross-contamination during culture; (iv) chromatography column contamination; (v) sequencing errors; and (vi) amino acid changes caused by RNA editing. Among these noise, PTM is one of the most influential factors in SAP detection. To reduce the false positive caused by PTM, we excluded 1908 SAP peptides whose corresponding wild-type peptides are identical to the PTM peptides in the EBI PRIDE clustering PTM dataset (14). Those peptides and their related information were displayed in the PTM webpage. We also compared the corresponding wild-type peptides of our variant peptides with the peptide sequences in EBI PRIDE clustering spectral library and found 6961 corresponding wild-type peptides in the EBI PRIDE library, which further support the reliability of our identified SAP peptides. We finally obtained 14 946 unique variant peptides and 1908 potential-PTM variant peptides (total 16 854 peptides) supported by 439 537 spectra. The number of peptides and proteins identified in each step of QC workflow is shown in Figure 2.

## DATABASE ACCESS AND DISPLAY

### dbSAP construction

We first conducted the identification of SAPs based on the MS data of 14 709 LC MS/MS experiments from diverse human tissues, cell lines and body fluids (Figure 1A). Then we built the SAP database (dbSAP) based on those identified single amino acid polymorphism peptides. The main purpose of dbSAP is to establish a reference spectral library regarding SAPs, and provide the associations among genes, variations, peptides/proteins and diseases. dbSAP mainly comprises four components corresponding to gene, variation, peptide and protein respectively, and users can search any component and navigate to the rest components through the links between them.

### Gene searching webpage

We collected gene-disease association information from various databases, including OMIM (current free available version) (32), PMD (22) and HPMD (23) as well as the information between genes and drugs from DrugBank (V5.0) (33) through mining the druggable genes. The associations between interested genes and related diseases, drug, pathways and variation information can be obtained by simply searching the database with gene symbol. Moreover, the gene symbols in the displayed table are also linked to the UCSC Genome Browser (http://genome.ucsc.edu/), which allows users to easily access any interested part of the genome. This webpage is also connected to related variation searching page with ‘Location’ link.

### Variation searching webpage

In the variation query page, if users perform the searching with dbSNP or COSMIC ID, dbSAP will return the associated gene, reference nucleotide and altered nucleotide. When user clicks the ‘dbSAP Accession’ and ‘Variation Peptide’ displayed in the ‘Single Amino Acid Polymorphism (SAP)’ table, a new webpage with corresponding protein and peptide information will be returned.

### Peptide searching webpage

The users can get the information of variants and variant related proteins and peptides in the peptide query webpage. When users enter the peptide sequence, dbSAP will return three tables: Peptide table, Spectrum table and Experiment table. In the Peptide table, users can get variant protein, peptide, reference Aa, altered Aa and SAP position in the peptide sequences. In the Spectrum table, dbSAP will retrieve the spectra of entry peptide when users click the spectrum icon. Users can also download all the spectra of the entry peptide through clicking ‘Download all’ button, or a single spectrum by clicking each ‘Spectrum ID’. In the experiment table, users can get the tissue name and experiment methods. When users put the mouse over the upper right corner of each table, the webpage will show the function of each button, such as ‘Hide/Show pagination’, ‘Refresh’, ‘Toggle’, ‘Columns’ and ‘Export data’. Users can download each table by clicking the ‘Export data’ button.

### Protein searching webpage

Users can also retrieve the information of SAPs and variant proteins from the protein searching webpage, including wild-type protein ID (Ensembl, Refseq and UniProt), dbSAP accession, SAP peptide in protein, variation position on the protein sequence, protein description and the source of the variation.

When users click ‘dbSAP Accession’, dbSAP will return three tables: protein table, protein sequence table and protein–protein interaction (PPI) table. In the ‘Protein Sequence’ table, the variant peptide and variant position in the protein sequence will be colored in red and light green and the Domain region in the protein sequence will be marked in yellow. Specifically, while users put the mouse on the SAP position in protein sequence, a picture of the stick structure of reference and altered Aa will be shown. Users can also get the PPIs derived from BioGRID database (3.4.138) (34) in the ‘PPI’ section. Moreover, when users click ‘Variation Peptide’ link, the webpage will jump to peptide search result webpage.

### PTM searching webpage

Post-translational modifications on amino acid residues may result in mass shift to the peptide spectra, such as Asn to Asp. We also collected those spectra and showed them in ‘PTM Page’. Users can view the potential PTM peptide and spectra by clicking the ‘Wild-type Peptide’ and ‘Variation Peptide’.

## DISCUSSION

Database searching is the most widely used method for protein identification, but has suffered from ‘the streetlight effect’ that only those peptides contained in the reference database are detectable. This effect may result in missing the meaningful information between SAPs and diseases, and hindering the understanding of the functional consequences of those protein-coding variants. However, the application of shotgun proteomics to identify the variant proteins remains a big challenge. Protein variants derived from nsSNPs and mutations largely depend on the known genomic variants, especially when the identified genomic variants and proteomics experiments are not matched.

To facilitate the functional study of variant proteins, we constructed dbSAP based on the large amount of MS data and a comprehensive set of human nsSNPs and mutations integrated from eight distinct databases. To better assess FDR, we introduced a strict QC workflow including global and group FDR strategies and we also reassembled the protein from peptides through QC criterion using a python script. Importantly, the associations between variant peptides and diseases were established according to the known associations between genes and diseases derived from OMIM, PMD and HPMD, which enables us to better understand the function of variant proteins. dbSAP enables related research communities to easily use the comprehensive reference dataset of variant proteins to conduct corresponding analyses.

We will continue to update dbSAP by collecting more MS data and SNPs/mutations to identify more SAPs. One of the important MS data resources is from the China Human Proteome Project (CNHPP) we are participating in (35,36), which will panoramically reveal human proteome in diverse tissues. CNHPP project will cover nine different cancers (including cancers of lung, gastric, esophageal and etc.), and the generated abundant data will lead us to catalog more disease related mutated peptides and spectra. Most spectra of the reference spectral libraries (such as NIST) are wild-type, dbSAP largely expends the reference spectral library and the detectable variation spectra, which provides a new perspective of protein-coding variants and could facilitate the studies of tumor pathogenesis and biomarker discovery. Collectively, dbSAP effectively connects nsSNPs/mutations, genes, peptides/proteins and diseases, which is valuable for interpreting the functions of SAPs in various human traits/diseases.

## References

[1] Abecasis G.R., Auton A., Brooks L.D., DePristo M.A., Durbin R.M., Handsaker R.E., Kang H.M., Marth G.T., McVean G.A., Genomes Project, C. (2012). An integrated map of genetic variation from 1,092 human genomes. Nature.

[2] Kidd J.M., Cooper G.M., Donahue W.F., Hayden H.S., Sampas N., Graves T., Hansen N., Teague B., Alkan C., Antonacci F. (2008). Mapping and sequencing of structural variation from eight human genomes. Nature.

[3] Chen G., Yang J., chen J., Song Y., Cao R., Shi T., Shi L. (2016). Identifying and annotating human bifunctional RNAs reveals their versatile functions. Sci China Life Sci.

[4] Saffen D. (2015). The genetic architecture of autism spectrum disorders (ASDs) and the potential importance of common regulatory genetic variants. Sci China Life Sci.

[5] Marth G.T., Yu F., Indap A.R., Garimella K., Gravel S., Leong W.F., Tyler-Smith C., Bainbridge M., Blackwell T., Zheng-Bradley X. (2011). The functional spectrum of low-frequency coding variation. Genome Biol..

[6] Di Fede G., Catania M., Morbin M., Rossi G., Suardi S., Mazzoleni G., Merlin M., Giovagnoli A.R., Prioni S., Erbetta A. (2009). A recessive mutation in the APP gene with dominant-negative effect on amyloidogenesis. Science.

[7] Alves P., Arnold R.J., Novotny M.V., Radivojac P., Reilly J.P., Tang H. (2007). Advancement in protein inference from shotgun proteomics using peptide detectability. Pac. Symp. Biocomput..

[8] O'Leary N.A., Wright M.W., Brister J.R., Ciufo S., Haddad D., McVeigh R., Rajput B., Robbertse B., Smith-White B., Ako-Adjei D. (2016). Reference sequence (RefSeq) database at NCBI: current status, taxonomic expansion, and functional annotation. Nucleic Acids Res..

[9] Karpova M.A., Karpov D.S., Ivanov M.V., Pyatnitskiy M.A., Chernobrovkin A.L., Lobas A.A., Lisitsa A.V., Archakov A.I., Gorshkov M.V., Moshkovskii S.A. (2014). Exome-driven characterization of the cancer cell lines at the proteome level: the NCI-60 case study. J. Proteome Res..

[10] Coordinators, N.R. (2016). Database resources of the National Center for Biotechnology Information. Nucleic Acids Res..

[11] Forbes S.A., Beare D., Gunasekaran P., Leung K., Bindal N., Boutselakis H., Ding M., Bamford S., Cole C., Ward S. (2015). COSMIC: exploring the world's knowledge of somatic mutations in human cancer. Nucleic Acids Res..

[12] Sheynkman G.M., Shortreed M.R., Frey B.L., Scalf M., Smith L.M. (2014). Large-scale mass spectrometric detection of variant peptides resulting from nonsynonymous nucleotide differences. J. Proteome Res..

[13] Zhang Z., Yang X., Mirokhin Y.A., Tchekhovskoi D.V., Ji W., Markey S.P., Roth J., Neta P., Hizal D.B., Bowen M.A. (2016). Interconversion of peptide mass spectral libraries derivatized with iTRAQ or TMT labels. J. Proteome Res..

[14] Griss J., Perez-Riverol Y., Lewis S., Tabb D.L., Dianes J.A., Del-Toro N., Rurik M., Walzer M.W., Kohlbacher O., Hermjakob H. (2016). Recognizing millions of consistently unidentified spectra across hundreds of shotgun proteomics datasets. Nat. Methods.

[15] Farrah T., Deutsch E.W., Hoopmann M.R., Hallows J.L., Sun Z., Huang C.Y., Moritz R.L. (2013). The state of the human proteome in 2012 as viewed through PeptideAtlas. J. Proteome Res..

[16] Perkins D.N., Pappin D.J., Creasy D.M., Cottrell J.S. (1999). Probability-based protein identification by searching sequence databases using mass spectrometry data. Electrophoresis.

[17] Gholami A.M., Hahne H., Wu Z., Auer F.J., Meng C., Wilhelm M., Kuster B. (2013). Global proteome analysis of the NCI-60 cell line panel. Cell Rep..

[18] Kim M.S., Pinto S.M., Getnet D., Nirujogi R.S., Manda S.S., Chaerkady R., Madugundu A.K., Kelkar D.S., Isserlin R., Jain S. (2014). A draft map of the human proteome. Nature.

[19] Pozniak Y., Balint-Lahat N., Rudolph J.D., Lindskog C., Katzir R., Avivi C., Ponten F., Ruppin E., Barshack I., Geiger T. (2016). System-wide clinical proteomics of breast cancer reveals global remodeling of tissue homeostasis. Cell Syst..

[20] Armandola E.A. (2003). Proteome profiling in body fluids and in cancer cell signaling. MedGenMed.

[21] Consortium U. (2015). UniProt: a hub for protein information. Nucleic Acids Res..

[22] Kawabata T., Ota M., Nishikawa K. (1999). The Protein Mutant Database. Nucleic Acids Res..

[23] Mathivanan S., Ji H., Tauro B.J., Chen Y.S., Simpson R.J. (2012). Identifying mutated proteins secreted by colon cancer cell lines using mass spectrometry. J. Proteomics.

[24] Li J., Duncan D.T., Zhang B. (2010). CanProVar: a human cancer proteome variation database. Hum. Mutat..

[25] Schandorff S., Olsen J.V., Bunkenborg J., Blagoev B., Zhang Y., Andersen J.S., Mann M. (2007). A mass spectrometry-friendly database for cSNP identification. Nat. Methods.

[26] Yates A., Akanni W., Amode M.R., Barrell D., Billis K., Carvalho-Silva D., Cummins C., Clapham P., Fitzgerald S., Gil L. (2016). Ensembl 2016. Nucleic Acids Res..

[27] Wang K., Li M., Hakonarson H. (2010). ANNOVAR: functional annotation of genetic variants from high-throughput sequencing data. Nucleic Acids Res..

[28] Brosch M., Yu L., Hubbard T., Choudhary J. (2009). Accurate and sensitive peptide identification with Mascot Percolator. J. Proteome Res..

[29] Kall L., Canterbury J.D., Weston J., Noble W.S., MacCoss M.J. (2007). Semi-supervised learning for peptide identification from shotgun proteomics datasets. Nat. Methods.

[30] Li J., Su Z., Ma Z.Q., Slebos R.J., Halvey P., Tabb D.L., Liebler D.C., Pao W., Zhang B. (2011). A bioinformatics workflow for variant peptide detection in shotgun proteomics. Mol. Cell. Proteomics.

[31] Fu Y., Qian X. (2014). Transferred subgroup false discovery rate for rare post-translational modifications detected by mass spectrometry. Mol. Cell. Proteomics.

[32] Amberger J.S., Bocchini C.A., Schiettecatte F., Scott A.F., Hamosh A. (2015). OMIM.org: Online Mendelian Inheritance in Man (OMIM(R)), an online catalog of human genes and genetic disorders. Nucleic Acids Res..

[33] Law V., Knox C., Djoumbou Y., Jewison T., Guo A.C., Liu Y., Maciejewski A., Arndt D., Wilson M., Neveu V. (2014). DrugBank 4.0: shedding new light on drug metabolism. Nucleic Aids Res..

[34] Oughtred R., Chatr-aryamontri A., Breitkreutz B.J., Chang C.S., Rust J.M., Theesfeld C.L., Heinicke S., Breitkreutz A., Chen D., Hirschman J. (2016). BioGRID: a resource for studying biological interactions in yeast. Cold Spring Harb. Protoc..

[35] Guerin M., Qian C., Zhong Q., Cui Q., Guo Y., Bei J., Shao J., Zhu X., Huang W., Wu J. (2016). Translational oncology toward benefiting cancer patients: the Sun Yat-sen University Cancer Center experience. Sci. China Life Sci..

[36] Tang H., Zhong F., Liu W., He F., Xie H. (2015). PathPPI: an integrated dataset of human pathways and protein-protein interactions. Sci. China Life Sci..

